# Differential roles of type I interferon signaling in tumor *versus* host cells in experimental glioma models^☆^

**DOI:** 10.1016/j.tranon.2022.101607

**Published:** 2022-12-24

**Authors:** Evelina Blomberg, Manuela Silginer, Patrick Roth, Michael Weller

**Affiliations:** a Laboratory of Molecular Neuro-Oncology, Department of Neurology, University of Zürich; b Laboratory of Molecular Neuro-Oncology*,* Department of Neurology, University Hospital Zurich, Zurich, Switzerland

**Keywords:** CRISPR/Cas9, Glioblastoma, Host, Model, Receptor, Signaling

## Abstract

Despite multimodal treatment approaches including surgery, radiotherapy and chemotherapy, the median survival for patients with glioblastoma remains in the range of one year and thus poor. Type I interferons (IFN) are involved in immune responses to viral infection and exhibit anti-tumor activity in certain cancers.

Here we explored the biological relevance of constitutive type I IFN signaling in murine glioma models *in vitro* and *in vivo*. CT-2A, GL-261, SMA-497, SMA-540 and SMA-560 murine glioma cells expressed IFN type I receptors IFNAR1 and IFNAR2 and were responsive to exogenous IFN stimulation. CRISPR/Cas9-mediated deletion of IFNAR1 decreased the baseline expression of type I IFN response genes in GL-261 cells, but neither in CT-2A nor in SMA-560 cells. IFNAR1 deletion slowed growth in GL-261 and SMA-560, but not in CT-2A cells. However, only the growth of IFNAR1-depleted GL-261 tumors and not that of SMA-560 tumors was delayed *in vivo* upon orthotopic tumor cell implantation into syngeneic mice. This survival gain was no longer detected when the IFNAR1-depleted GL-261 cells were inoculated into IFNAR1-deficient mice. Altogether these data suggest that constitutive type I IFN signaling in gliomas may be pro-tumorigenic, but only in a microenvironment that is proficient for type I IFN signaling in the host.

## Highlights

• IFNAR1 knockout decreased proliferation *in vitro* in SMA-560 and GL-261 glioma cells.
• GL-261 was the only model demonstrating constitutive type 1 IFN signalling *in vitro*.
• Only GL-261 IFNAR1 knockout conferred survival benefit *in vivo*.
• IFNAR1 knockout-conferred survival benefit depends on intact IFNAR1 host signalling.

## Introduction

Gliomas are primary tumors of the central nervous system and are classified by their histopathological features and molecular genetic alterations. Glioblastoma, WHO grade 4 glioma, is an aggressive tumor characterized by invasiveness, focal necrosis, and microvascular proliferation [1]. Patients diagnosed with glioblastoma have a poor prognosis with a median survival around 12 months in epidemiological studies [2] and 16 months in clinical trial populations [3]. Standard of care treatment comprises surgical resection as safely feasible followed by radiotherapy with concomitant and maintenance temozolomide chemotherapy [4].

Interferons (IFN) are multifunctional cytokines which can activate and modulate both innate and adaptive immunity. IFN are divided into three families depending on which receptors they activate. The type I IFN family consists of approximately 20 members, namely IFN-α, IFN-β, IFN-ε, IFN-κ, IFN-ω, IFN-δ, IFN-ζ, and IFN-τ, with IFN-α and IFN-β being most important in the context of immune activation. Classical type I IFN signaling is induced upon type I IFN binding to the two IFNAR subunits, IFNAR1 and IFNAR2. IFNAR1 has low binding affinity for type I IFN ligands, but induces intracellular signaling while IFNAR2 contributes to stabilization of ligand binding by high binding affinity [5]. When type I IFN bind to the receptor, the two subunits heterodimerize and autophosporylation of the intracellular kinase domains takes place. IFN-β is additionally known to signal through IFNAR1 homodimers independent of IFNAR2 [6]. The canonical signaling pathway leads to phosphorylation of different signal transducer and activator of transcription (STAT) transcription factors. Phosphorylation of STAT prompts homo- or heterodimerization of different STAT family members such as STAT1 and STAT3. This in turn induces nuclear translocation where the pSTAT homo- or heterodimers bind interferon-stimulated response elements (ISRE) or gamma activated sequences (GAS) [7]. pSTAT binding to ISRE together with other IFN-related regulatory factors leads to subsequent transcription of IFN-stimulated genes.

Type I IFNs have been mainly characterized as a barrier against viral infection, but have also attracted interest as immunotherapeutic agents in a variety of human cancers, including pancreatic cancer, melanoma, renal cell carcinoma, and various liquid neoplasia [8,9].

Autocrine type I IFN signaling has been associated with various pro-tumorigenic features. IFN signaling promotes expression of the immunosupressive immune checkpoint molecule, programmed death ligand-1 (PD-L1) [10]. Mutations in the IFN signaling pathway may be prognostic for response to immune checkpoint inhibitors in melanoma [11]. Furthermore, constitutive type I IFN downstream signaling, *e.g.*, via pSTAT1, correlates with invasiveness of breast cancer in humans as well as shorter survival of mice implanted with mouse mammary carcinoma cells overexpressing an active form of STAT1 [12]. Constitutive STAT1 overexpression has been associated with radioresistance in squamous carcinoma and melanoma cells *in vivo* [13,14]. Beyond radioresistance, STAT1 overexpression has been associated with chemoresistance in lymphocytic leukemia and ovarian cancer cells [15,16]. We have previously studied the potential role of type I IFN signaling in glioma models. We detected IFNAR1 and IFNAR2 mRNA and protein and mRNA of ligands IFN-α and IFN-β in all human glioma cell lines tested. We also obtained evidence that type I IFN signaling is active in gliomas *in vivo*. By analyzing Tumor Cancer Genome Atlas (TCGA) RNA-seq data we confirmed that expression of the IFN response gene, MxA, was higher in gliomas than in normal brain, with glioblastomas demonstrating the highest expression. This finding was also confirmed by immunohistochemistry. When the TCGA cohort was divided into MxA high and low cohorts, low expression was associated with better survival, consistent with a contribution of type I IFN signaling to glioma agressiveness [17]. Here we explored the importance of constitutive type I IFN signaling in the tumor and in the microenvironment in syngeneic immunocompetent murine glioma models.

## Materials and methods

### Cell lines

CT-2A and GL-261 murine glioma cell lines were purchased from Merck/Millipore (Millipore Cat# SCC194, RRID:CVCL_ZJ44) and the National Cancer Institute (Frederick, MD) respectively. SMA-497, SMA-540 and SMA-560 cells were provided by Dr. D. Bigner (Duke, NC). The cells were cultured in Dulbecco's Modified Eagle Medium (DMEM) supplemented with 2 mM L–glutamine and 10% fetal bovine serum (FBS) (Gibco Life Technologies, Zug, Switzerland).

### Reagents

Recombinant murine IFN-β1a was generously provided by Biogen (Cambridge, MA). Murine IFN-γ was purchased from PeproTech (Rocky Hill, NJ).

### CRISPR/Cas9 genome editing

The MIT optimized CRISPR design tool (http://crispr.mit.edu/) was used to design two *Ifnar1* targeting single guide RNA (sgRNA) sequences. The following sequences were used: 5′- ATGTTCCCGTCTTGTCCGGG-3′ and 5′-AGACTTCTGCCAGATTCGTA-3. SgRNA sequences were synthesized by Microsynth (Balgach, Switzerland) and cloned into pSpCas9-GFP [18]. The pSpCas9 (BB)−2A-GFP (PX458) plasmid, which contains green fluorescent protein (GFP) and Cas9, was a kind gift from Dr. F. Zhang (RRID:Addgene_48138, Teddington, UK). TransIT-X2 (Mirus Bio, Madison, WI) transfection agent was used to introduce the final constructs into the glioma cells. Single cell colonies were expanded and screened for targeted DNA disruption by reverse transcriptase quantitative polymerase chain reaction (RT-qPCR) with primers aligning to the cut sites and by verifying the absence of protein by flow cytometry.

### Density-dependent proliferation assay

Proliferation in different seeding densities was assessed by seeding cells in sextuplicates of serial dilutions ranging from 128 to one cell/well in 96-well plates. After 10 days the cell cultures were stained with crystal violet (C2886, Sigma) for 10 min and the absorbance was quantified at 562 nm (Infinite M200 Pro-plate-reader, Tecan Life Sciences, Männedorf, Switzerland).

### RT-qPCR

RNA was extracted by a column system (NucleoSpin, Macherey-Nagel AG, Oensingen, Switzerland) and transcribed into cDNA by a cDNA Reverse Transcription Kit (Applied Biosystems by Thermo Fisher Scientific, Waltham, MA). Primers were: Hprt1: forward 5′-TTGCTGACCTGCTGGATTAC-3′, reverse 5′-TTTATGTCCCCCGTTGACTG-3′, mouse Ifnar1: forward 5′-GCCCAAGGCAAGAGCTATGT-3′, reverse 5′-TTGAATAGCCAGGAAGCCACT-3′, mouse Ifnar2: forward 5′-GACGAAAATCTGACGAAGGTTAAGA-3′, reverse 5′- ATTATTTTGGAAGTGACAGGTGGAA-3′, mouse Ifnar1 for CRISPR validation: forward 5′-ACATCTCTCCTCCCGGACAA-3′, reverse 5′-TCTGCCAGATTCGTATGGTGT-3′, mouse Mx1: forward 5′-CCTGGTAGGACAGCTCTT-3′, reverse 5′-GAACTGCTGCCAAGGAAG-3′. PowerUp SYBR Green master mix (Applied Biosystems) was used to quantify gene expression in a QuantStudio 6 Flex Real-Time PCR system machine (Applied Biosystems). The cycles consisted of the following steps: 95 °C 15 s, 60 °C 1 min, repeated 40 times. Relative gene expression was calculated with the 2 ^(-ΔCT)^ method. *Hprt1* was used for normalization [19].

### Immunoblot analysis

The cells were lysed with radioimmunoprecipitation assay lysis buffer (10x, 10 mM Tris pH 8.0, 150 mM NaCl, 1% NP-40, 0.5% deoxycholate, 0.1% sodium dodecyl sulfate, Merck Millipore, Burlington, MA) supplemented with phosphatase inhibitor cocktails 2 (P5726) and 3 (P0044) and protease inhibitor (P8340) (Sigma-Aldrich, St. Louis, MO). Protein concentration was assessed by Pierce BCA protein assay kit (Thermo Fisher Scientific). Protein (25 µg per lane) was boiled in Laemmli buffer containing 10% β-mercaptoethanol (Sigma-Aldrich), separated by sodium dodecyl sulfate polyacrylamide gel electrophoresis (SDS-PAGE) and transferred to polyvinylidene fluoride (PVDF) membranes (GE Healthcare, Buckinghamshire, UK). Horseradish peroxidase (HRP)-coupled secondary antibodies (Santa Cruz Biotechnology Cat# sc-2357, RRID:AB_628497, 1:5000) were used for detection together with enhanced chemiluminescence (Pierce, Thermo Fisher Scientific). Primary antibodies were diluted 1:1000 in 5% skim milk (Rapilait, Migros, Switzerland) or 5% bovine serum albumin (BSA) (AppliChem, Darmstadt, Germany) and used as follows: anti-pSTAT1 rabbit anti-mouse (Cell Signaling Technology Cat# 9167, RRID:AB_561284), anti-STAT1 rabbit anti-mouse (Cell Signaling Technology Cat# 9172, RRID:AB_2198300). Equal loading was ensured by staining with an HRP-conjugated actin antibody (Santa Cruz Biotechnology Cat# sc-47778, RRID:AB_626632).

### Flow cytometry

Accutase (Thermo Fisher Scientific) was used to detach the cells, to preserve surface protein levels. After cell count determination, the cells were stained with fluorescently labeled antibodies together with a viability dye as specified below at 4 °C for 30 min. Stained cells were washed and resuspended in flow cytometry buffer (1x PBS, 5% FBS, 2 mM EDTA, 2 mM NaN_3_), analyzed by BD FACSVerse flow cytometer (BD Biosciences) and quantified with FlowJo (RRID:SCR_008520,TreeStar, Ashland, OR). Protein levels were quantified as specific fluorescence indexes (SFI) by dividing median fluorescence of experimental staining and isotype staining. The following antibodies and dyes were used: APC anti-mouse IFNAR1 antibody (BioLegend Cat# 127313, RRID:AB_2122746, 1:200) and APC mouse IgG1, κ isotype control (BD Biosciences Cat# 550854, RRID:AB_398467), Zombie Aqua viability dye (1:1000, BioLegend, San Diego, CA).

### Animal experiments

C57BL/6 Ifnar^−/-^ mice (RRID:MMRRC_032045-JAX) were kindly provided by Dr. B. Becher (Zurich, Switzerland). Ifnar1^−/−^ and VM/Dk mice (RRID:IMSR_HAR:618) were bred at the University of Zurich in pathogen-free laboratories. C57BL/6 wild-type mice (MGI Cat# 2159769,RRID:MGI:2159769) were obtained from Janvier Labs (Le Genest-Saint-Isle, France). Surgery was performed at age 6–12 weeks; 100,000 GL-261 cells or 20,000 SMA-560 cells were implanted into the right striatum of female and male syngeneic mice. Anesthesia (fentanyl 1 mL (=0.05 mg/kg, midazolam (Dormicum, Basel, Switzerland) 1 mL (=5.00 mg/kg), medetomidin (Magny-Vernois, France) 0.5 mL (=0.5 mg/kg) was injected subcutaneous prior to tumor inoculation. In addition was analgesia given for pain relief, carprofen (Rimadyl, Parsippany-Troy Hills Township, NJ, USA) 0.1 mL (=0.17 mg/30 g) + 2.9 mL aqua ad 3 mL á 0.1 mL/30 g bodyweight. Mice were unblinded randomized into groups (*n* = 10, 7 for survival and 3 for histology) receiving either parental or modified cells. The mice were routinely checked for neurological symptoms and their weight was monitored. Euthanasia was performed when developing score 2 neurological symptoms. These include weight loss more than 15% compared to weight at tumor inoculation, light left leg paralysis, moderate pain indications or decreased/no activity. Prior to euthanasia by cervical dislocation, mice were anesthetized by 4% isoflurane gas (Attane, Provet AG, Lyssach, Switzerland). All animal studies’ standard operating procedures were approved by Swiss Cantonal Veterinary office under animal license ZH098/2018 and ZH109/2020.

### Histology

Brains were collected at time of euthanasia and snap-frozen in Chryochrome (Epredia, Shandon, NH). For further staining, brains were cut in 8 μm thick sections and thereafter mounted on histological glass slides. The tissue was fixed for 10 min in 4% formaldehyde solution (Fisher Chemical, Thermo Fisher Scientific) and thereafter treated for 10 min with hydrogenperoxide solution of 3% (Merck Millipore). Prior to staining the slides were blocked with SuperBlock (ScyTek Laboratories, West Logan, UT) for 30 min at room temperature and then incubated with primary antibodies at 4 °C overnight. Primary antibodies were anti-Ki67 (Acris Antibodies Cat# DRM004, RRID:AB_1004358), anti-CD3 (BD Biosciences Cat# 555273, RRID:AB_395697), anti-CD11b (BD Biosciences Cat# 550282, RRID:AB_393577) and anti-CD45 (BioLegend Cat# 103102, RRID:AB_312967). The slides were then stained with corresponding pre-labeled secondary antibodies from Histofine Simple Stain MAX PO (Nichirei, Tokyo, Japan). The staining was visualized by 3, 3′-diaminobenzidine (DAB) (ImmPACT DAB staining kit, Vector Laboratories, Burlingame, CA). Mayer`s Hematoxylin (JT Baker, Biosystems, Muttenz, Switzerland) was used for 1 min counterstaining of the nucleus. After dehydration, the slides were mounted with Rotimount medium (Carl Roth). Axio Scope.A1 light microscope (Carl Zeiss, Oberkochen, Germany) was used to take photographs. DAB staining intensity was determined by ImageJ on 2–3 fields per tumor at 10x magnification.

### TCGA analysis

R2 microarray analysis and visualization platform (http://r2.amc.nl) was used to visualize survival analysis of TCGA data. Kaplan-Meier curves were created by selecting a target gene and using the median scan cutoff mode. The 540–MAS5.0–u133a glioblastoma dataset (*n* = 540) was used.

### Statistical analysis

Graphs depict representative experiments in which mean and standard deviation (SD) are shown where applicable. GraphPad Prism 8 (RRID:SCR_002798, GraphPad Software, San Diego, CA) was used for statistical testing. For experiments with two groups, unpaired t-tests assuming equal SD were used and for experiments with more than two groups and paired samples, one-way or two-way ANOVA were used. Additionally, Tukey's post hoc test was used to adjust for multiple testing. Survival data are depicted as Kaplan-Meier survival curves and log-rank tests were used to determine significance. Significance was symbolized as *p<0.05, **p<0.01, or ***p<0.001.

## Results

### Mouse glioma cells express IFN-β and type I IFN receptors

**Fig. 1:**
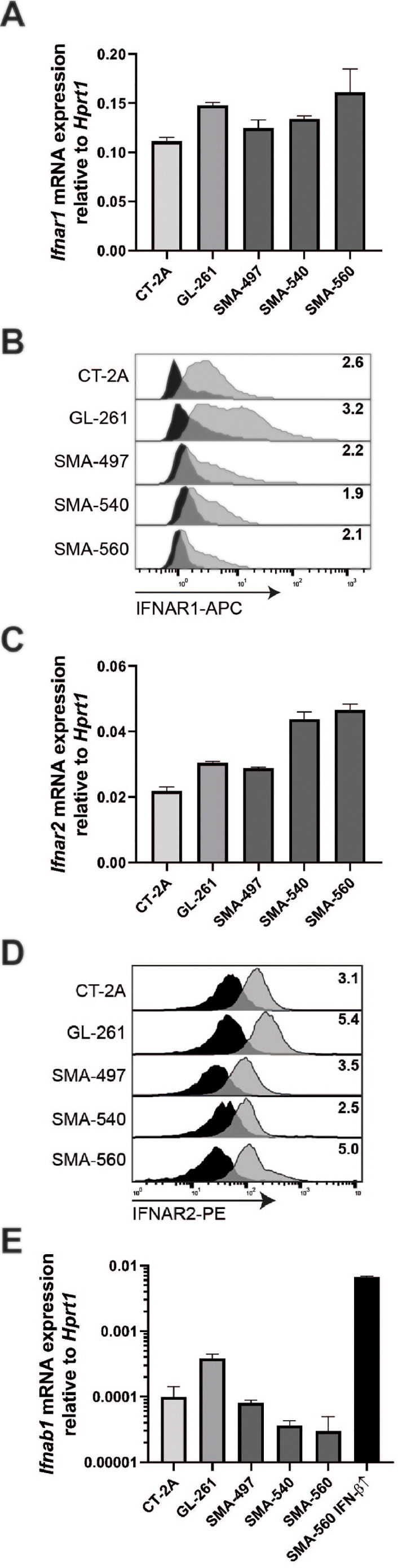
**Expression of type I IFN receptors and ligands in mouse glioma cells *in vitro*.** Relative *Ifnar1, Ifnar2* and *Ifnb1a* mRNA expression was assessed by RT-qPCR using *Hprt1* as a reference gene and SMA-560 *Ifnb1a* over-expressing cells as a positive control. Data are expressed as mean and SD (A,C,E). IFNAR1 and IFNAR2 protein levels were determined by flow cytometry. Black histograms represent isotype control antibody staining whereas gray histograms represents experimental antibody staining. Protein levels were quantified by SFI values normalized to an isotype control antibody and presented as numerical values in the graph (B,D).

All five mouse glioma cell lines uniformly expressed mRNA for both type I IFN receptors, *Ifnar1* and *Ifnar2* (Fig. 1A,C). Surface protein levels were determined by flow cytometry (Fig. 1B,D). There was no close correlation between mRNA and protein levels. Low mRNA levels of ligand *Ifnab1* were detected in all cell lines (Fig. 1E)*.* Only SMA-560 and GL-261 showed amplification of *Ifn1a* mRNA, but at detection levels close to the reliability threshold (data not shown).

**Fig. 2:**
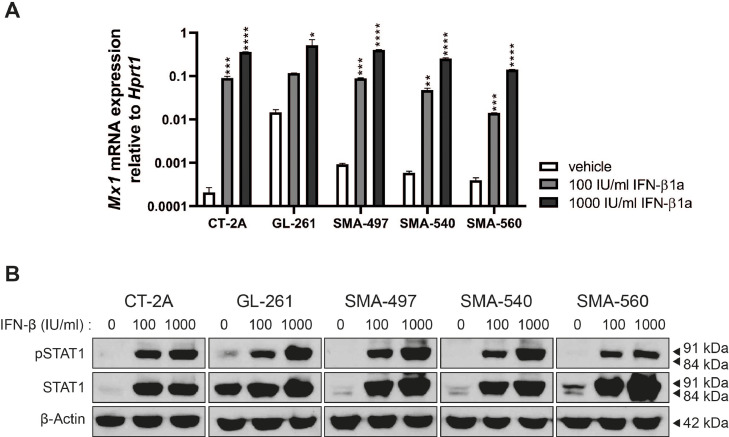
**Activation of classical type I IFN signaling in mouse glioma cells *in vitro***. Responsiveness to IFN-β1a was assessed at the level of *Mx1* mRNA expression by RT-qPCR (A) and quantification of STAT1 phosphorylation and total STAT1 protein levels by immunoblot (B) after exposure to the indicated concentrations of IFN-β1 for 24 h. In these experiments, *Hprt1* was used as a reference gene for RT-qPCR and actin was used as loading control for immunoblots. Data are represented as mean and SD (two-way ANOVA with Dunnett's post hoc test for multiple comparison,*p<0.05, **p<0.01, ***p<0.001, ****p<0.0001).

To demonstrate that the type 1 IFN signaling pathway is intact, exogenous IFN-β was added to the cells and downstream interferon stimulated gene induction was determined. The cells were responsive in a concentration-dependent manner to exogenous IFN-β as evidenced by the induction of *Mx1* mRNA expression and the phosphorylation of STAT1. High basal levels of Mx1 mRNA, STAT1 protein and phosphorylated STAT1 were only seen in GL-261 (Fig. 2A,B), suggesting constitutive autocrine IFN signaling in these cell. This signaling is independent on exogenous IFN-β, but consistent with the highest endogenous IFN-β production (Fig. 1E).

### Generation and characterization of IFNAR1-deficient glioma cell lines

**Fig. 3:**
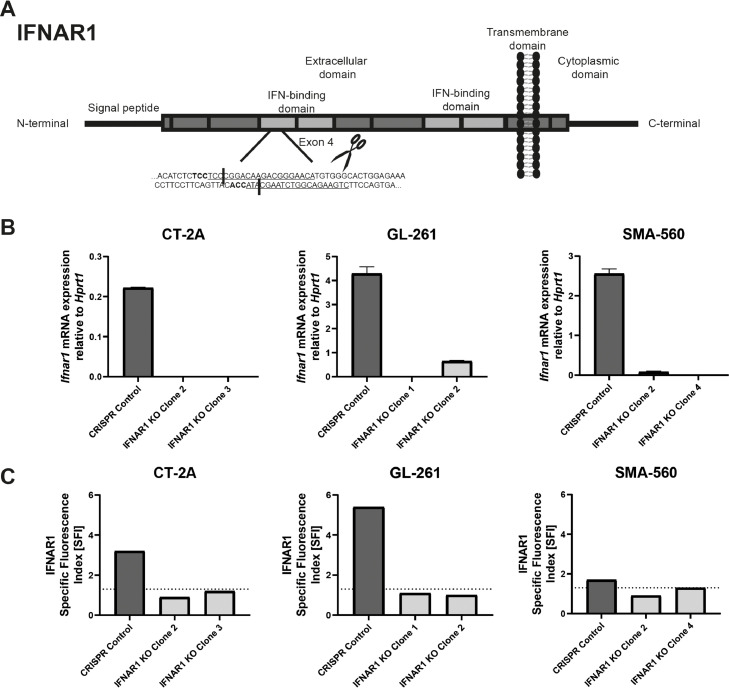
**Generation of IFNAR1 CRISPR/Cas9 knockout sublines.** A. Schematic overview of target sites of sgRNA sequences on *Ifnar1* gene which were targeted. B. Specific primers overlapping predicted cut sites of sgRNA were used to verify gene disruption by RT-qPCR. C. IFNAR1 protein levels were assessed by flow cytometry. Protein quantification is represented by SFI values which are normalized to an isotype control antibody. Threshold value of 1.3, demonstrated by dotted line, indicate lack of expression.

To further investigate the function of IFNAR1 in mouse glioma models, we generated glioma sublines deficient for *Ifnar1* using CRISPR/Cas9-mediated knockout. Two sgRNA were designed that create double stranded DNA cuts in the IFN binding domain of IFNAR1, making the receptor non-functional (Fig. 3A). The sgRNA were cloned into a vector containing Cas9 and GFP and then transfected into the indicated cell lines. CRISPR control cells were generated by transfecting cells with two sgRNA without a specific target. GFP-positive single cells were sorted in 96-well plates for clonal expansion and subsequent screening to obtain sublines with complete knockout of *Ifnar1*. Primers aligning to the predicted cut sites were designed to be able to determine successful disruption of *Ifnar1* by RT-qPCR. If the double stranded DNA break occurred, the primers would not be able to align to the sequence and no amplification would take place. None or very little amplification was seen in CT-2A, GL-261 and SMA-560 knockout clones (Fig. 3B). Protein levels were determined by flow cytometry and clones which were negative or had very low surface protein level were chosen for further characterization (Fig. 3C).

**Fig. 4:**
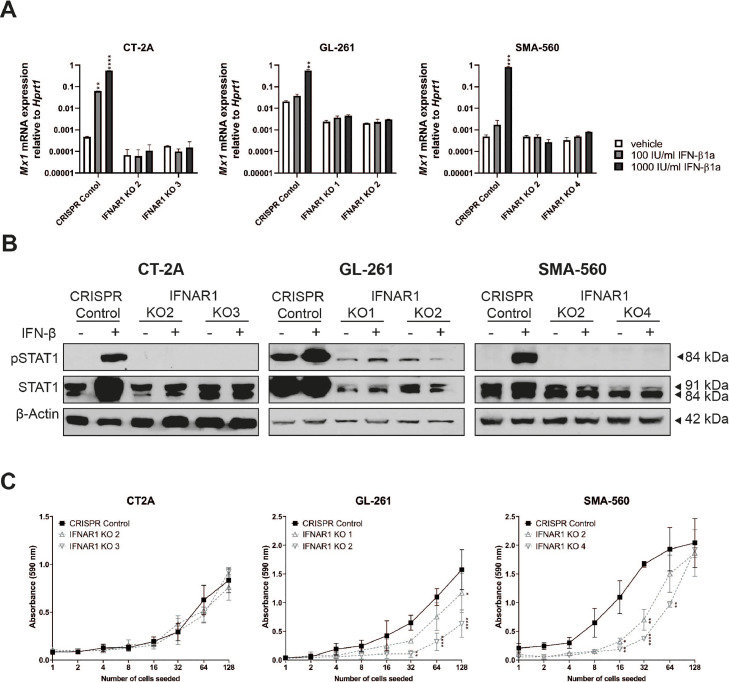
**Abrogation of type I IFN receptor signaling upon IFNAR1 knockout.** A. *Mx1* mRNA expression was assessed by RT-qPCR after exposure to IFN-β1a (100–1′000 U/ml) for 24 h using *Hprt1* as a reference gene. Data are represented as mean and SD (two-way ANOVA with Dunnett's post hoc test for multiple comparison, **p<0.01, ***p<0.001, ****p<0.0001). B. Phosphorylation of STAT1 and total STAT1 protein levels were assessed by immunoblot using actin as a loading control. Where indicated, cells were treated with IFN-β1a (1000 U/ml) for 24 h. C. CRISPR control or IFNAR1 KO cells were seeded at increasing densities in 96-well plates and incubated for 7–10 days to determine cell growth. At the endpoint, the wells were stained with crystal violet and absorbance was measured at 590 nm (two-way ANOVA with Dunnett's post hoc test for multiple comparison,*p<0.05, **p<0.01, ****p<0.0001).

The absence of functional IFNAR1 was verified by the abrogation of *Mx1* induction and STAT1 phosphorylation in response to IFN-β. Only CRISPR control cells showed upregulation upon IFN-β stimulation whereas levels remained unchanged in IFNAR1 knockout cells (Fig. 4A,B). The constitutively decreased *Mx1* mRNA and pSTAT1 and STAT1 protein levels in IFNAR1-deficient GL-261 cells confirm autocrine control of these genes by type I IFN in this cell line.

The cell growth of these sublines was investigated by means of a proliferation assay with serial seeding densities. By seeding cells in serial dilution, also below standard seeding densities, growth and clonogenic survival in sub-optimal conditions were determined. SMA-560 and GL-261 IFNAR1 knockout cells showed decreased growth while the growth of CT-2A IFNAR1 knockout cells was unaffected in the proliferation assays (Fig. 4C).

### Effects of IFNAR1 deficiency in glioma cells *in vivo*

**Fig. 5:**
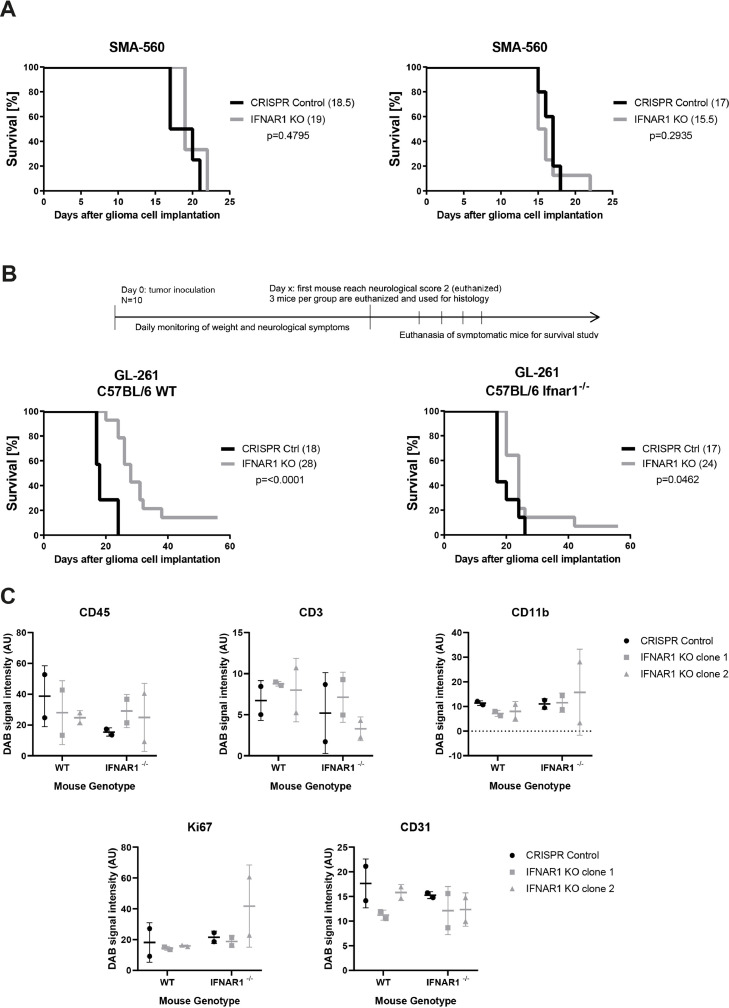
**IFNAR1 knockout in the host, but not in tumor cells, affects the survival of tumor-bearing mice.** A.SMA-560 CRISPR Control or IFNAR1 KO cells were orthotopically implanted in VM/Dk mice. Two biological replicates are demonstrated. B. Schematic overview of timeline of the *in vivo* experiments and subsequent histological examination. GL-261 CRISPR Control or IFNAR1 KO cells were orthotopically implanted in C57BL/6 wild-type (left) or *Ifnar1^−/^*^−^ mice (right). Survival data are presented as Kaplan–Meier plots. (log-rank test,’*p<0.01, ***p<0.001). Median survival is indicated within brackets in graph legends. C. Quantitative analysis of immunohistochemical stainings of advanced tumors from Fig. 5B. Tumor-specific staining was quantified by DAB intensity of 2–3 fields at 10x magnification. Each dot represents one tumor respectively one mouse.

Because of GL-261′s high basal expression and SMA-560 high responsiveness to IFN-β stimulation, GL-261 and SMA-560 CRISPR control or IFNAR1 knockout cells respectively were orthotopically implanted in syngeneic mice. In contrast to the *in vitro* data (Fig. 4C), the growth of tumors formed by SMA-560 IFNAR1-depleted cells was unaffected (Fig. 5A). Conversely, when GL-261 IFNAR1 knockout cells were implanted in mice, there was a significant survival benefit compared to mice implanted with control cells (Fig. 5B, left). To investigate the specific role of type I IFN signaling in the host, wild-type or IFNAR1 KO cells were implanted into IFNAR1-deficient mice. The survival benefit associated with IFNAR1 knockout in GL-261 cells was abrogated when these cells were implanted into IFNAR1-deficient mice (Fig. 5B, right). To elucidate possible explanations for the survival difference seen in wild-type mice, histological examination of advanced tumors was performed. Proliferation, vascularization and immune infiltration was examined by staining for Ki67, CD31, CD45, CD3, and CD11b. No significant change between control or IFNAR1 KO cells implanted in wild-type or IFNAR1^−/−^ mice was observed (Figs. 5C, S1).

## Discussion

Because of its suggested central role in bridging innate and adaptive immunity, type I IFN signaling is considered an important pathway to study in the context of cancer immunotherapy. Intratumoral administration of IFN-β gene therapy showed great efficacy in promoting rejection of GL-261 tumors in mice [20] and therapeutic concentrations of IFN-β sensitize glioma cells including glioma stem-like cells to irradiation and temozolomide [21,22]. Prolonged exposure to exogenous IFN-β induced a fragile glioblastoma stem cell phenotype with a transcriptional profile of reduced migratory and mitogen-activated protein kinase pathway activity [23]. The current study aimed to improve our understanding of autocrine and paracrine IFNAR signaling in glioma cells and its microenvironment.

We have previously reported that numerous human glioma cell lines exhibit constitutive expression of type I IFN-induced genes. The disruption of IFN signaling enhanced sensitivity to natural killer cell-mediated lysis as well as decreased expression of PD-L1 protein at the cell surface. In addition, we confirmed its relevance *in vivo* by demonstrating increased expression of the type 1 IFN response gene, MxA, in glioblastoma and its association with inferior survival in the TCGA dataset [17]. Furthermore, two type I IFN single nucleotide polymorphisms have been associated with poorer survival in glioma patients [24] and TCGA analysis revealed an association with inferior survival in patients with tumors with high IFNAR1 RNA expression. No survival association was observed for high *versus* low RNA expression of ligands IFNA1 and IFNB1 (Fig. S2). However, many isoforms of type I IFN exist in humans, therefore, looking at IFN response gene signatures might be more relevant. Several studies have outlined that high expression of IFN response signatures correlates with poorer survival in glioblastoma patients in both TCGA and Chinese Glioma Genome Atlas (CGGA) cohorts [25,26].

Multiple clinical trials have investigated the addition of type I IFN to the standard of care. Either IFN-α [27] or IFN-β [28] failed to prolong survival in recurrent glioma cohorts. IFN-β additionally failed to afford any clinical benefit in newly diagnosed glioblastoma patients [29]. A newer strategy of delivering IFN to glioma tumors has also been studied. A phase I trial investigated whether gene transfer by intratumoral injection of hIFNb could be safely executed however, all patients progressed within 4 months after treatment [30].

To fully understand the mechanism by which type I IFN signaling may contribute to glioma progression, immunocompetent models need to be used, notably since IFN is a known immune regulator. Here, we explored the role of type 1 IFN signaling in syngeneic mouse glioma models. We find that all mouse glioma cell lines expressed type I IFN receptors and were responsive to exogenous IFN-β. However, only GL-261 showed constitutive expression of IFN-β and IFN-β pathway activation (Figs. 1,2). To understand the role of autocrine or paracrine type I IFN signaling in murine glioma models *in vitro* and *in vivo*, we utilized CRISPR/Cas9 to disrupt the *Ifnar1* gene. IFNAR1 knockout cells were no longer susceptible to exogenous IFN-β. Consistent with the observation that GL-261 had the highest baseline type I IFN signaling activity, abrogating receptor expression had an effect on baseline IFN-stimulated gene expression in GL-261, but not in CT-2A or SMA-560 cells. GL-261 showed reduced proliferation when IFNAR1 was abrogated. The same was true in SMA-560 cells although this cell line did not appear to have measurable autocrine type I IFN signaling under standard culture conditions (Fig. 4). Both cell lines with reduced growth *in vitro* upon *Ifnar1* gene disruption were orthotopically implanted *in vivo*. SMA-560 showed no phenotype, whereas the GL-261 model showed increased survival of mice when IFNAR1 signaling in the tumor was abrogated (Fig. 5A-B). In the SMA-560 model, the host may provide growth-promoting cues to IFNAR1-deficient glioma cells that are not available *in vitro*. Theoretically, the abrogation of the growth advantage of IFNAR1-proficient parental SMA-560 cells could also be explained by a growth inhibitory effect of host-derived IFN-β on the control tumors, but not on the IFNAR1-deficient tumors.

The consequences of IFNAR1 depletion have been studied in other tumor entities. IFNAR1 depletion did not alter the tumorigenicity *in vivo* in colon adenocarcinoma, melanoma, pancreatic ductal adenocarcinoma or Lewis lung carcinoma models. However, all four IFNAR1 knockout tumor models acquired radiosensitivity demonstrated by significantly prolonged survival *in vivo* [31]. Furthermore, the acquired radiosensitivity of the colon adenocarcinoma IFNAR1 knockout cells depended on the presence of CD8 T effector cells. In the current study, IFNAR1 depletion resulted in a phenotype *in vivo* only in the glioma model that exhibited constitutive type 1 IFN signaling. It suggests that merely blocking IFN signaling is not sufficient to alter growth *in vivo*, but only when the cells have constitutive signaling at baseline are the cells sensitive to the IFN signaling disruption caused by IFNAR1 depletion. Others have reported that genetic abrogation of constitutive STAT3 signaling, a downstream molecule of IFN signaling, leads to increased release of inflammatory cytokines by human glioblastoma cells which in turn increased dendritic cell maturation [32], Moreover, pharmacological inhibition of constitutive STAT3 signaling decreased proliferation of U251 human glioblastoma cells *in vitro* and increased overall survival in the mouse glioma model Tu-9648 *in vivo* [33,34]. One proposed mechanism of action of these studies is reduced proliferation, however, we observed reduced proliferation only *in vitro*, but not *in vivo* (Figs. 4C, 5C).

Interestingly, when IFNAR1-depleted GL-261 cells were implanted into the brains of IFNAR1-deficient mice, the survival gain was abrogated, indicating that this effect was dependent on intact type I IFN signaling in the host (Fig. 5B). Accordingly, we propose that blocking constitutive type I IFN signaling in glioma cells may abrogate cues of the microenvironment that enhance the tumorigenicity of some glioma models. In a microenvironment deficient of type I IFN signaling, this pathway appears no longer operational, suggesting some crosstalk of type I IFN signaling between tumor and host cells.

### Financial support

The study was supported by a grant from the 10.13039/501100001711Swiss National Science Foundation (310030_185155 / 1), the DETAS foundation and by the Clinical Research Priority Program (CRPP) of the 10.13039/501100006447University of Zurich for the CRPP ImmunoCure.

### Data availability statement

The data that was generated in this study are available upon request from the corresponding author.

### Authorship statement

Experimental design and implementation (E.B., M.S., and M.W.), analysis, interpretation of the data and reviewing the manuscript (E.B., M.S., P.R and M.W), writing of the original manuscript draft (E.B., M.W.), funding acquisition (P.R, M.W), supervision (M.S and M.W).

## Declaration of Competing Interests

The authors declare that they have no known competing financial interests or personal relationships that could have appeared to influence the work reported in this paper.

EB reports no conflicts of interest.

MS reports no conflicts of interest.

PR has received honoraria for lectures or advisory board participation from Bristol-Myers Squibb, Boehringer Ingelheim, Debiopharm, Merck Sharp and Dohme, Novocure, QED, and Roche and research support from 10.13039/100009947Merck Sharp and Dohme and Novocure.

MW has received research grants from Apogenix, Merck, Sharp & Dohme, Merck (EMD), Philogen and Quercis, and honoraria for lectures or advisory board participation or consulting from Adastra, Bayer, Bristol Meyer Squibb, Medac, Merck, Sharp & Dohme, Merck (EMD), Nerviano Medical Sciences, Novartis, Orbus, Philogen and y-Mabs.

## Appendix Supplementary materials

Image, image 1

Image, image 2
