# Supplementary figures and images for: Differential roles of type I interferon signaling in tumor versus host cells in experimental glioma models
Source: Transl Oncol. 2022 Dec 24;28:101607. doi: 10.1016/j.tranon.2022.101607 (PMC9800198; doi:10.1016/j.tranon.2022.101607)

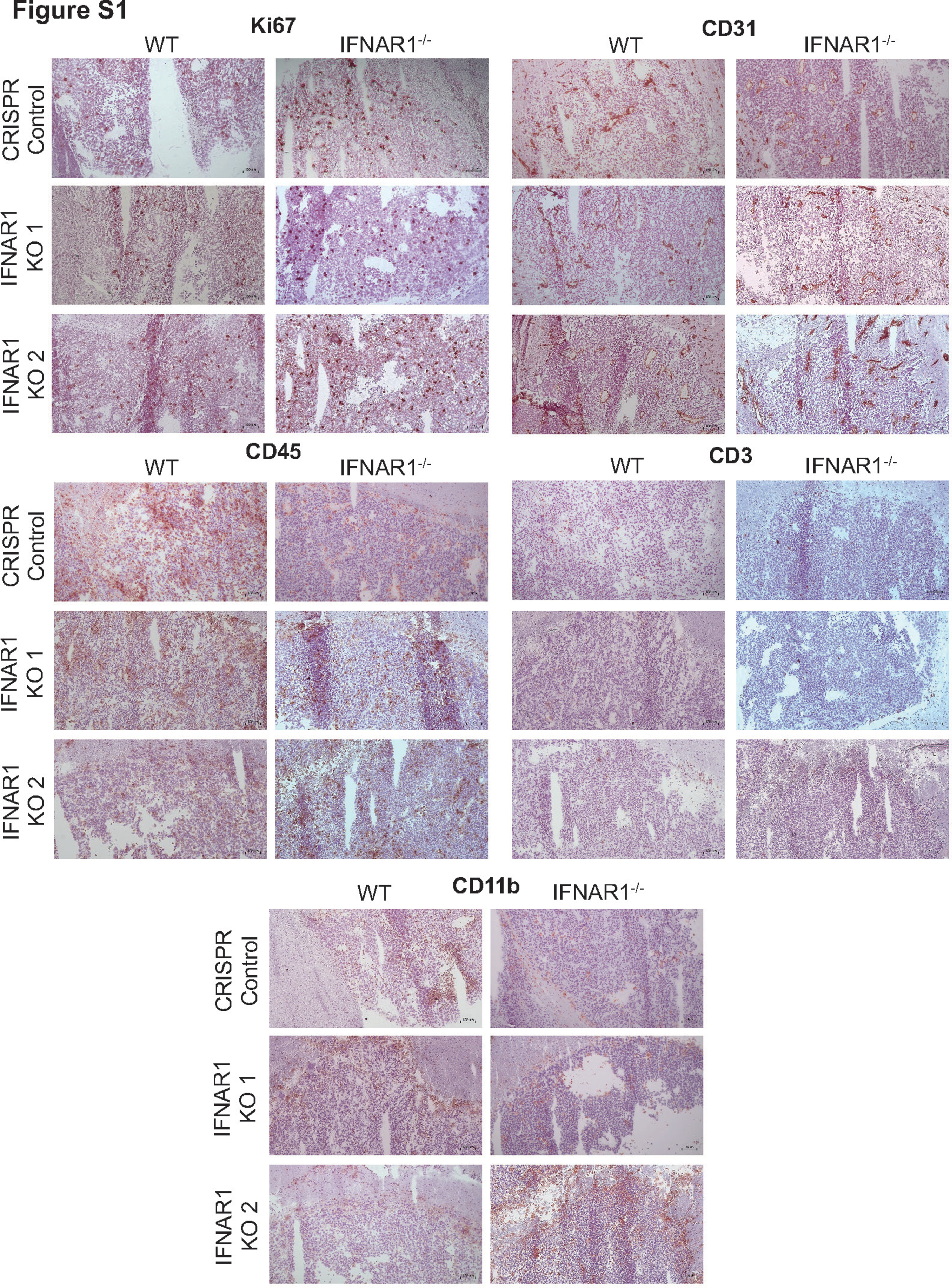

Supplement: Supplementary file 1 [file mmc1.jpg]

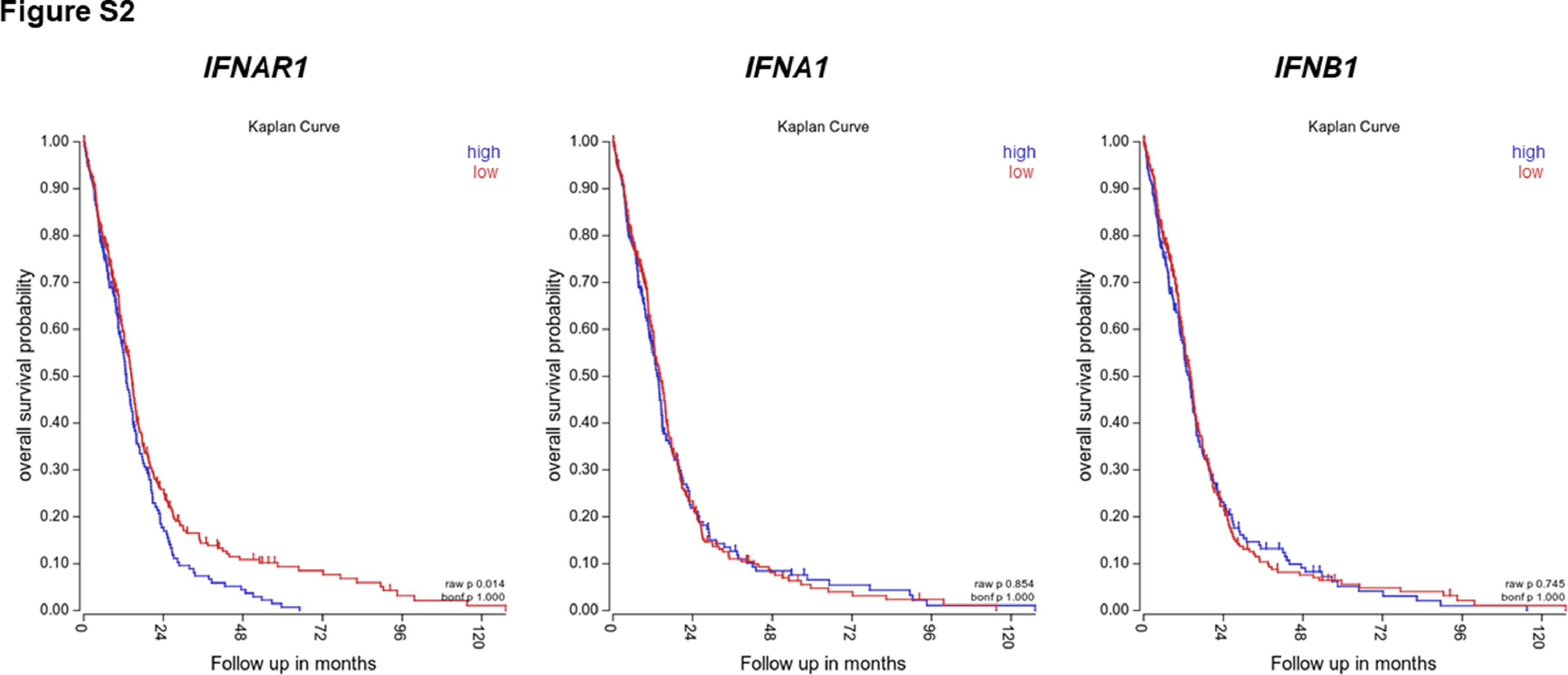

Supplement: Supplementary file 2 [file mmc2.jpg]
